# A Microfluidic Device for Culturing an Encapsulated Ovarian Follicle

**DOI:** 10.3390/mi8110335

**Published:** 2017-11-20

**Authors:** Aziz Ur Rehman Aziz, Mengjie Fu, Jiu Deng, Chunyang Geng, Yong Luo, Bingcheng Lin, Xiaohui Yu, Bo Liu

**Affiliations:** 1 Department of Biomedical Engineering, Dalian University of Technology, Dalian 116024, China; azizjatoi@hotmail.com (A.U.R.A.); 1352257919@mail.dlut.edu.cn (C.G.); 2 Dalian Institute of Maternal and Child Health Care, Dalian 116024, China; fmjnewlife@126.com; 3 School of Pharmaceutical Science and Technology, Dalian University of Technology, Dalian 116024, China; dengjiu@mail.dlut.edu.cn (J.D.); yluo@dlut.edu.cn (Y.L.); bclin@dicp.ac.cn (Bi.L.)

**Keywords:** ovarian follicles, microfluidics, organ on a chip

## Abstract

Microfluidic chips have been proved effective in mimicking different organs of human body. Simulating human ovarian follicles by microfluidic device will be useful in exploring the mechanism of folliculogenesis and related diseases. In this paper, a microfluidic chip was designed to culture a single human pre-antral follicle. Ovarian follicles were first encapsulated in 3D calcium alginate hydrogel beads and then cultured on chip and in dish under same conditions. The diameters of cultured ovarian follicles were measured, and the same amount of medium was collected from microfluidic device or dish per two days for measuring the estradiol and androgen concentrations. The results confirmed the successful growth of ovarian follicles on chip with their hormonal trends and diameters increase, which were similar to ovarian follicles cultured in dish. It is concluded that this microfluidic chip can be used to culture a single human ovarian follicle, which provides a useful tool to explore the hormonal changes and their interactions during folliculogenesis.

## 1. Introduction

Several organs have been successfully cultured on chip and their diseased models have also been presented with microfluidic devices [1]. For simulation of an organ on a chip, it is necessary to develop a functional unit on the chip, which can summarize the whole function of the organ [1]. Ovarian follicle (OF) is the functional unit of an ovary where an immature egg develops and produces the reproductive hormones such as estrogen and progesterone [2]. A single OF is required to be cultured on a chip in an ovary-like microenvironment for an ovary simulation. Earlier, two-dimensional (2D) and three-dimensional (3D) approaches have been used for in vitro culture of OFs [3]. For 2D culture on dish surface, theca cells and granulosa cells detach, spread out and attach on the surface leading to non-physiological morphology [4]. Endogenous autocrine and paracrine factors from both types of cells are diluted, which affect the OF development [5]. In 3D approaches with millimeter-sized alginate encapsulation, the OF architecture is preserved efficiently [6,7,8]. Folliculogenesis, the growth of OF, depends on different hormones. Androgen is the major hormone in the regulation of primary follicles to secondary follicles, and then to pre-antral follicle, while estradiol (E2) is the major hormone in the transition of pre-antral follicles to antral follicles [9]. A microfluidic device [10] was presented for the miniaturized 3D culture of secondary OFs of deer mice, with alginate and collagen to fabricate cortical and medullar tissues of ovary. This device facilitated the OFs development but it was not used to culture OFs in biomimetic ovarian tissue. Instead, the microtissues were cultured in cell culture plates and culture medium was collected for hormone analysis [11]. Recently, another microfluidic device [12] was designed which supported the groups of murine OFs to produce human menstrual cycle (28 days) hormone profile. Ten OFs were encapsulated in one alginate hydrogel bead and cultured in a microfluidic system and hormone levels were measured [12]. However, a group of mice OFs hormone data cannot express the hormone profile of single human OF.

Hormones are very crucial factors in the development of OF and some disturbance in their concentrations may cause different ovarian diseases. The hormone released from a single human OF can provide the constructive information for understanding the folliculogenesis and conducting its clinical trials. Therefore, there is a strong demand for a device to collect hormones and simulate some diseased models of a single growing human OF. In this paper, a microfluidic device was designed for the first time to culture a single human OF encapsulated in 3D calcium alginate hydrogel bead to analyze its hormone secretions. Similar behavior was observed in the hormone secretions of OFs in a dish under the same conditions.

## 2. Materials and Methods

### 2.1. Isolation of Ovarian Follicles

**Figure 1:**
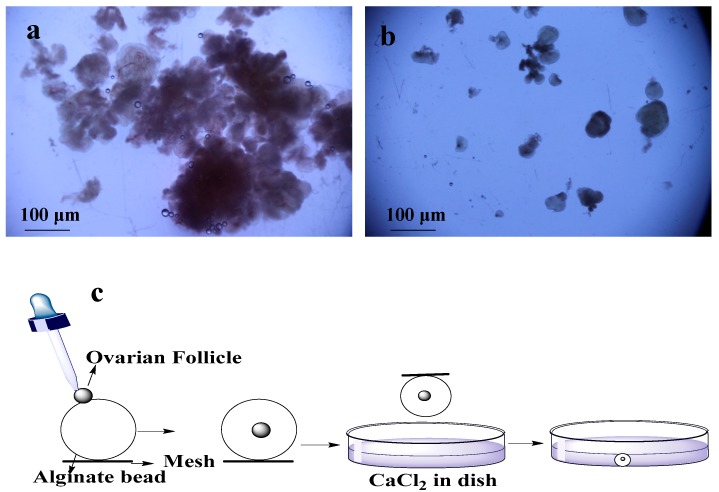
Isolation and encapsulation of ovarian follicles (OFs): (**a**) tissues used for enzymatic digestion for isolation of OFs; (**b**) mechanically isolated OFs; (**c**) encapsulation of OFs by using alginate bead and CaCl_2_.

Ovarian biopsies were taken from Dalian maternity hospital with ethical approval. These isolated tissues were transferred to lab within 2 h in a bottle of cold (4 °C) 20 mL transfer medium (10% fetal bovine serum (FBS) (Sigma-Aldrich, St. Louis, MI, USA) + 1% Penicillin/Streptomycin solution (PS) + 89% minimum essential medium (MEM)-α (1X) + GlutaMAX™-1 (Gibco^®^, Thermo Fisher Scientific, Waltham, MA, USA). Phosphate buffer saline (PBS) was used to wash the tissues two to three times, and most of the medullar tissues and blood vessels were removed in dissection medium (MEM-α (1X) + GlutaMAX™-1 +1% FBS + 1% PS) by scalpel. The cortical tissues were cut into ~1 mm^3^ pieces by surgical scissors. First pre-antral follicles were isolated by using 25-guage needles mechanically (Figure 1a) and remaining tissues were digested with enzymes (0.2% Collagenase type IA (Solarbio, Life Sciences, Beijing, China) and 0.02% DNase I (Solarbio, Life Sciences, Beijing, China) [13]. For digestion, remaining pieces of tissues were transferred into 50 mL conical flask containing 20 mL PBS supplemented with collagenase (1 mg/mL). The volume of collagenase solution was two times of the tissues. Then, incubation was done for 1 h at 37 °C in water bath with shaking the flask and tissues after every 15 min to mechanically disrupt the digested tissues. An equal volume of PBS medium (pre-cooled in the refrigerator at 4 °C) supplemented with 10% FBS was added to terminate the digestion. This resulting suspension was centrifuged at 4 °C and 50 g for 10 min [14]. After discarding the supernatant, two times the volume of freshly prepared dissection medium was added in the centrifuging tube and centrifuged again. After transferring the pellets on dish with new dissection medium, pre-antral follicles were mechanically isolated using 25-gauge needles under microscope [15] (Figure 1b).

### 2.2. Encapsulation of Ovarian Follicles

For encapsulation of the OFs, 0.5% of sodium alginate (Sigma-Aldrich, St. Louis, MI, USA) solution was made of sodium alginate, and 2.0% of calcium chloride (Sigma-Aldrich, St. Louis, MI, USA) solution was made of calcium chloride. These solutions were disinfected at 120 °C for 30 min [16]. A single OF was transferred to individual drop of alginate with pipette, and then this alginate bead was placed in the encapsulation solution of calcium chloride for 2 min (Figure 1c). After encapsulation, bead was washed with MEM-α (1X) medium for CaCl_2_ removal [14,17]. 20 OFs were encapsulated and half of them were cultured in dishes and remaining half were cultured on chips.

### 2.3. Culture of Ovarian Follicle in Dish

Each encapsulated OF was placed in separate well of the 24 well plate containing 400 μL of culture medium (50% MEM-α (1X) + GlutaMAX™-1, 50% F-12 (1X) + GlutaMAX™-1, FBS (1 mg/mL), 5 μg/mL insulin (Solarbio, Life Sciences, Beijing, China), 5 μg/mL transferrin human (Sigma-Aldrich, St. Louis, MI, USA), 5 μg/mL sodium selenite (Sigma-Aldrich, St. Louis, MI, USA), BSA (3 mg/mL), 1:100 recombinant follicle stimulating hormone (rFSH) [18]. This 24 well plate was placed in an incubator at 37 °C with 95% relative humidity and 5% CO_2_. Culture medium (400 μL) was collected after every ~48 h and fresh 400 μL was added for eight days when the diameters of OFs were ~400–500 μm [18]. Two hundred μL of collected culture medium was used to test the hormones.

### 2.4. Fabrication of the Microfluidic Model

**Figure 2:**
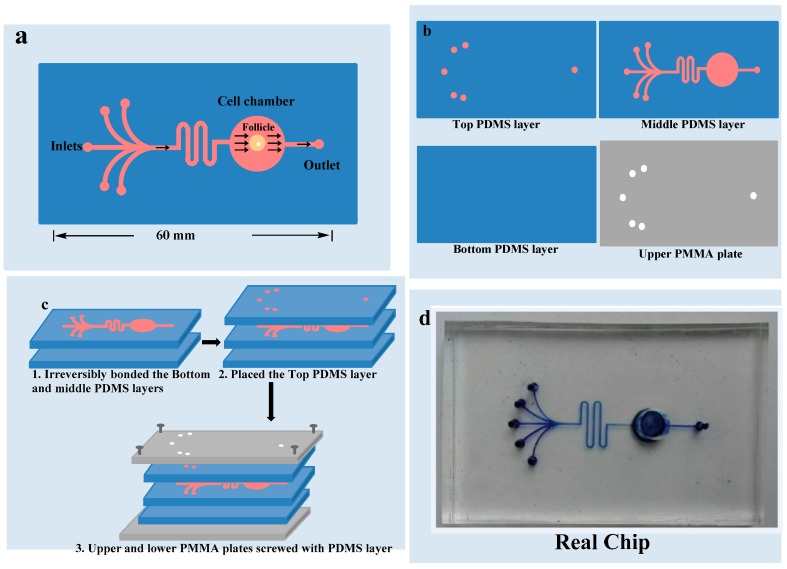
Chip design: (**a**) 5 inlets and 1 outlet with one chamber where OF is cultured; (**b**) 3 polydimethylsiloxane layers and upper polymethyl methacrylate layer; (**c**) All the layers combined together; (**d**) Real chip.

As demonstrated in Figure 2, microfluidic device was composed of two plates of polymethyl methacrylate (PMMA) substrate and three layers of a polydimethylsiloxane (PDMS) plates. Heights of the upper, middle and lower PDMS layers were 2 mm, 5 mm and 1 mm respectively. Upper layer of PDMS had one circular outlet and five inlets points of 1.5 mm diameters for culture medium. Middle PDMS layer had channels of 100 μm widths and 160 μm heights for medium flow, and a circular culture chamber of 6 mm diameter and 5 mm height. Lower PDMS layer did not have any channel or chamber. Middle PDMS layer was fabricated by repeating the molding of the masters, prepared by the spin coating of a 100 μm wide and 160 μm thick layer of SU8-3035 negative photo-resist (Microchem Corp., Newton, CA, USA) on a silicon wafer and then patterned by photolithography respectively. The Sylgard-184 PDMS base and curing agent (Dow Corning, Midland, MI, USA) were mixed with the ratio of 10:1 by mass thoroughly and degassed under vacuum and then poured on masters. The polymer was cured in an oven for 1 h at 80 °C. After cooling, the PDMS layers were gently peeled off from the masters and finally trimmed to their sizes [19]. Holes of inlets and outlet were created in the upper PDMS layer for introduction of the medium. The culture chamber was manufactured with a hole in the middle PDMS layer before bonding it with the bottom PDMS layer. Middle and lower PDMS layers were irreversibly bonded together by the treatment of oxygen plasma for 1 min. The upper PMMA plate was perforated at the places of inlets and outlet in advance. Microfluidic device was ultraviolet (UV) sterilized for 2 h before use. First channels were filled with medium and then OF was placed carefully in the culture chamber to avoid bubble formation. The upper PDMS layer was placed to close the chamber. After careful alignment along the vertical direction, all of the three PDMS layers were placed over each other with the top and bottom PMMA plates and fastened with screws. The entire microfluidic device measured 6 cm × 4 cm × 0.8 cm.

### 2.5. Culture of Ovarian Follicle on Chip

**Figure 3:**
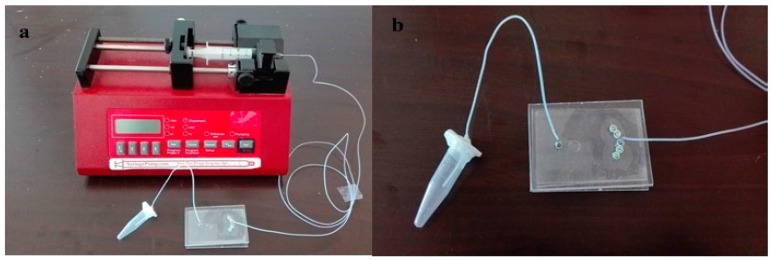
Microfluidic apparatus: (**a**) Microfluidic device showing the pump, chip and collecting tube arrangement; (**b**) chip with collecting tube.

An encapsulated OF was cultured in the chamber of the microfluidic chip. This device was placed in an incubator at 37 °C with 95% relative humidity and 5% CO_2_. Cultural medium was provided to the chip by a syringe pump from inlet which was controlled by an automated system (8.33 μL/h). After 48 h, medium was collected from the outlet into a 1.5 mL tube and stored at −20 °C (Figure 3). 200 μL of collected culture medium was used for hormone testing.

### 2.6. Follicle Measurements and Hormone Tests

Photographs of each OF were captured during culture period by light microscope linked with a camera. 17β-estradiol (E2) and Testosterone (T) concentrations were measured by enzyme-linked immunosorbent assay (ELISA) with Elecsys Estradiol III assay kit and Elecsys Testosterone II assay kit (Roche Diagnostics, Basel, Switzerland) respectively, as instructed by the manufacturers. All the assays were processed in duplicate and mediums collected from the wells and tubes without OFs were used as negative controls.

### 2.7. Statistical Analysis

OFs diameters and hormone secretions were analyzed by *t*-test and significance level was set at (*p* < 0.05).

## 3. Results

### 3.1. Diameter of the Follicles

**Figure 4:**
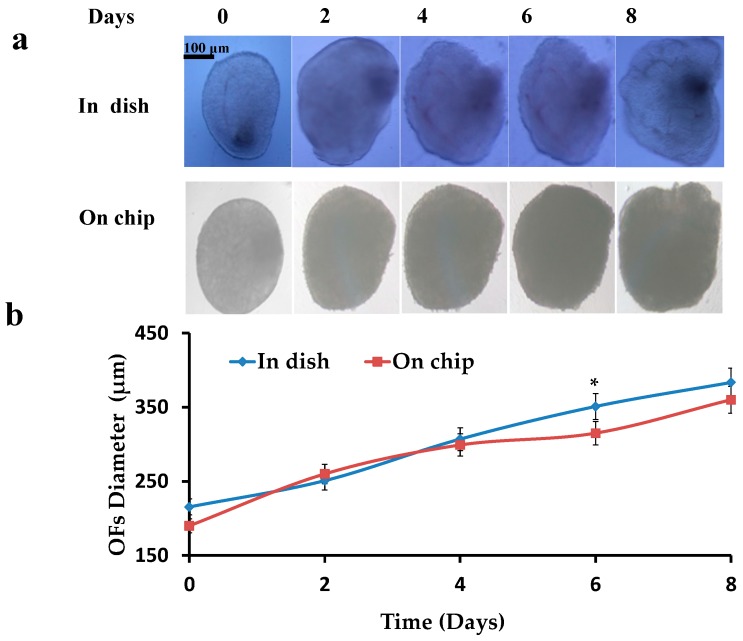
Increase in diameter of OFs: (**a**) Cultured OFs in dish and on chip; (**b**) Graphical representation of increase in diameters of OFs in dish and on chip.

Pre-antral OFs were isolated, encapsulated (0.5% alginate hydrogels) and cultured in dish and on chip, and then the increase in diameters of OFs was investigated. Initial diameters of the OFs were 190 ± 30 μm. OFs were cultured for 8–10 days till the formation of the pre-ovulatory OFs. A constant increase (*p* < 0.05 compared to the start diameter) in the diameters of OFs was observed ranging to 380 ± 30 μm (Figure 4), while most of the time points showed no difference between the OFs in dish and on chip (*p* > 0.05).

### 3.2. Hormone Tests for the Ovarian Follicles in Dish and on Chip

**Figure 5:**
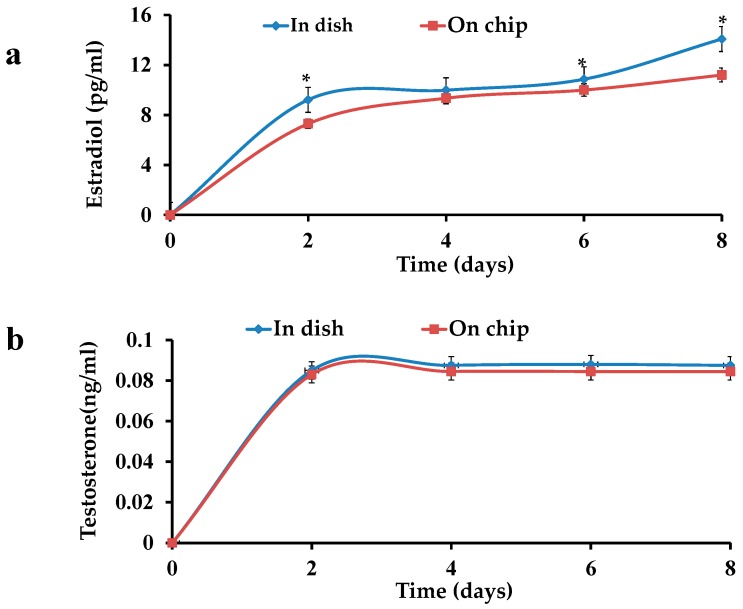
Hormone tests of OFs: (**a**) increase in estradiol level in a dish and on a chip; (**b**) Testtosterone in a dish and on chip.

E2 and testosterone levels were examined in the culture medium of the OFs. An increase in the E2 concentration was observed in both OFs i.e., in dish and on chip. On the other hand, T levels almost remained constant in both cases. A difference (*p* < 0.05) in the E2 values of OFs in dish and on chip was observed, while the same type of increasing trend was seen in the both cultures (Figure 5a). No significance difference (*p* < 0.05) in the T values of both cultures was observed (Figure 5b).

## 4. Discussion

Folliculogenesis mainly depends upon the hormones, and their disturbance can cause abnormal follicle growth which leads to different ovarian diseases such as polycystic ovarian syndrome. Hence, provision of a precise dynamic environment to a growing follicle is crucial for understanding the hormone secretions during its growth and exploring different diseases and their proper treatments. Earlier, OFs were cultured in dish and tested for their hormone levels [18] where they required more nutrients and cost. It is very hard to provide a precise biomimetic environment and other dynamic conditions to the developing OFs in dish. These shortcomings can be solved by using a microfluidic system. Microfluidic devices have been used for the simulation of many organs on chip, including OFs. Choi et al. designed a microfluidic device and cultured OFs from deer mice (Peromyscus), but that device was not used for hormone data collection [10]. They encapsulated OFs in alginate and collagen beads to fabricate the ovarian cortical and medullary tissues. That encapsulation is more complex than simple calcium alginate hydrogel encapsulation. Their device contained more outlets without collecting tube or testing kit. Instead, the medium was collected from culture plates for hormone analysis [11]. Recently, another microfluidic device has been designed that facilitated the groups of OFs culture of CD-1 female mice that were encapsulated in an alginate hydrogel bead. The culture produced the hormone profile that controls the female human reproductive tract and peripheral tissues [12]. Culturing of OFs group is easier than a single OF culture, and mice follicles cannot be the behalf of human follicles. Culturing of OFs in groups cannot provide the data of a single OF. In addition, it would not be reliable to count numbers of follicles and try to evaluate the data of a single follicle, because there must be any interaction or interference among the groups of follicles. Here another point should also be noted that in a human usually there is only one follicle that reaches maturation stage at one time, which means OFs grow independently.

To simulate an ovary and provide a tool for a single OF hormone analysis, a microfluidic device has been designed for culturing a single human OF encapsulated in calcium alginate hydrogel on a chip in this project for the first time. This microfluidic device is simpler and can also be used to collect hormones data from the cultured OF. More inlets are present in this chip to provide different culture mediums for the growing follicle and supply different hormones or factors to check their influences on its growth. By changing concentrations of the provided hormones/factors to the OF, some related diseases can be simulated within this model to analyze the effects of different drugs on a growing follicle/diseased follicle. This microfluidic device could be helpful in exploring the interactions among the hormones and also useful in studying the factors affecting the hormone expressions. Chamber size is designed according to the size of OFs that can be used to culture OFs of every stage. However, in this project, hormone data from OFs cultured in a dish and on a chip were compared. The data from the diameters and hormones expressions of the OFs were consistent with previous literature [8,20]. It proved the successful OF culture on chip like dish.

Granulosa cells synthesize E2 by aromatase, which is expressed in small follicles of foetal granulosa cells [20] but expression is low till birth. This expression first increases in pre-antral follicles and small antral follicles in neonatal period depending upon gonadotropins and follicle stimulating hormone (FSH) [21].Then it decreases in small follicles and pre-antral follicles, however only remaining in large antral follicles during childhood [20]. At the antral follicles stage, FSH promotes granulosa cells differentiation functions e.g., synthesize E2. Therefore, androgens role and their interactions are no longer applicable while E2 role is more important till terminal follicle maturation. In this study, constant androgen levels represent no interactions with E2, as E2 is increasing continuously, while the literature proved the depression of anti-mullerian hormone (AMH) at these stages suggesting no interaction of androgen and AMH [9]. These results support the idea of no interaction and less role of androgen at these stages. In vivo data suggests repression of AMH by E2. Gonadotropin recovery causes the E2 rise during pubertal transition and slight decrease of AMH level without direct inhibition of FSH [22,23,24]. Stimulated ovaries for in vitro fertilization show AMH decrease especially on human chorionic gonadotropin (hCG) triggering day and suggested negative correlation between serum AMH [25,26,27] and E2. Lee represented a relative increase of E2 and a decrease of AMH from the beginning of stimulation to hCG [27], and the same results were seen in follicle fluids of mature follicles [28,29,30]. This relation was not seen in follicular phases of other cycles [25]. Liberty also challenged the repression of AMH by E2 while comparing the AMH level in different situations [31]. Recently, a decrease in AMH was also mentioned by Gnoth et al. from five days before ovulation to two days after ovulation [32], which also showed the same relationship in vitro studies. There was a response element of E2 AMH gene [33]. E2 shows AMH stimulatory effect for AMH via estrogen receptor (ER) Alpha, the main receptor in corpus luteum, and an inhibitory effect via ER beta [34], which is important receptor in granulosa cells [35]. From above data it can be concluded that E2 inhibits AMH during the late follicular phase overcoming the FSH stimulatory effect. AMH is higher in the absence/low expression of E2 in the early follicular phase. So E2 secretion which depends upon FSH is found in the relation with FSH and AMH [9]. Our experimental results also support the above conclusions, as the increase in the E2 values with the diameter increase of the OFs represents the important role of E2 in these stages. A difference was seen in E2 values of both cultures, which could be due to the different sizes of the chambers of dish and chip. It could also be because of the continuous medium flow in the microfluidic device, while in dish, the medium was stationary. The smaller size of the chamber and the continuous flow of the medium provided the shear stress and more in vivo-like environment in chip. This device is more reliable than the dish because nutrients provision is continuous and more controlled. The microfluidics chip provides a more reliable microbiomimetic environment as compared to the dish. However, further research is needed to explore the mechanism of folliculogenesis and related diseases with this device.

## 5. Conclusions

Our model of the chip has been successfully used to culture a single human OF and report the hormones in second stage of folliculogenesis. This model can provide an easy approach to apply various factors to growing OF for exploring the changes in its structure and hormone production. By changing the hormones and other related factors, diseased models can be developed and it could be helpful in drug screening tests. This device provides a tool that could be helpful in understanding the role of hormones in growing OF, which can reveal different aspects of follicular growth and several pathophysiological conditions.

## References

[1] Aziz A.U.R., Geng C., Fu M., Yu X., Qin K., Liu B. (2017). The role of microfluidics for organ on chip simulations. Bioengineering.

[2] He X.M., Toth T.L. (2017). In vitro culture of ovarian follicles from peromyscus. Semin. Cell Dev. Biol..

[3] Desai N., Alex A., AbdelHafez F., Calabro A., Goldfarb J., Fleischman A., Falcone T. (2010). Three-dimensional in vitro follicle growth: Overview of culture models, biomaterials, design parameters and future directions. Reprod. Biol. Endocrinol..

[4] Choi J.K., Agarwal P., He X. (2013). In vitro culture of early secondary preantral follicles in hanging drop of ovarian cell-conditioned medium to obtain mii oocytes from outbred deer mice. Tissue Eng. Part A.

[5] West E.R., Shea L.D., Woodruff T.K. (2007). Engineering the follicle microenvironment. Semin. Reprod. Med..

[6] Shikanov A., Xu M., Woodruff T.K., Shea L.D. (2009). Interpenetrating fibrin-alginate matrices for in vitro ovarian follicle development. Biomaterials.

[7] Shea L.D., Woodruff T.K., Shikanov A. (2014). Bioengineering the ovarian follicle microenvironment. Annu. Rev. Biomed. Eng..

[8] He X. (2017). Microfluidic encapsulation of ovarian follicles for 3d culture. Ann. Biomed Eng..

[9] Dewailly D., Robin G., Peigne M., Decanter C., Pigny P., Catteau-Jonard S. (2016). Interactions between androgens, fsh, anti-mullerian hormone and estradiol during folliculogenesis in the human normal and polycystic ovary. Hum. Reprod. Update.

[10] Choi J.K., Agarwal P., Huang H., Zhao S., He X. (2014). The crucial role of mechanical heterogeneity in regulating follicle development and ovulation with engineered ovarian microtissue. Biomaterials.

[11] Agarwal P., Choi J.K., Huang H., Zhao S., Dumbleton J., Li J., He X. (2015). A biomimetic core-shell platform for miniaturized 3d cell and tissue engineering. Part. Part. Syst. Charact..

[12] Xiao S., Coppeta J.R., Rogers H.B., Isenberg B.C., Zhu J., Olalekan S.A., McKinnon K.E., Dokic D., Rashedi A.S., Haisenleder D.J. (2017). A microfluidic culture model of the human reproductive tract and 28-day menstrual cycle. Nat. Commun..

[13] Xu M., Kreeger P.K., Shea L.D., Woodruff T.K. (2006). Tissue-engineered follicles produce live, fertile offspring. Tissue Eng..

[14] Xu M., Barrett S.L., West-Farrell E., Kondapalli L.A., Kiesewetter S.E., Shea L.D., Woodruff T.K. (2009). In vitro grown human ovarian follicles from cancer patients support oocyte growth. Hum. Reprod..

[15] Roy S.K., Greenwald G.S. (1996). Methods of separation and in vitro culture of pre-antral follicles from mammalian ovaries. Hum. Reprod. Update.

[16] Camboni A., Van Langendonckt A., Donnez J., Vanacker J., Dolmans M.M., Amorim C.A. (2013). Alginate beads as a tool to handle, cryopreserve and culture isolated human primordial/primary follicles. Cryobiology.

[17] Xu M., Banc A., Woodruff T.K., Shea L.D. (2009). Secondary follicle growth and oocyte maturation by culture in alginate hydrogel following cryopreservation of the ovary or individual follicles. Biotechnol. Bioeng..

[18] Xiao S., Zhang J., Romero M.M., Smith K.N., Shea L.D., Woodruff T.K. (2015). In vitro follicle growth supports human oocyte meiotic maturation. Sci. Rep..

[19] Jin D., Ma X.C., Luo Y., Fang S.M., Xie Z.R., Li X.J., Qi D.Y., Zhang F.Y., Kong J., Li J. (2016). Application of a microfluidic-based perivascular tumor model for testing drug sensitivity in head and neck cancers and toxicity in endothelium. RSC Adv..

[20] Stocco C. (2008). Aromatase expression in the ovary: Hormonal and molecular regulation. Steroids.

[21] Gray S.A., Mannan M.A., O’Shaughnessy P.J. (1995). Development of cytochrome p450 aromatase mrna levels and enzyme activity in ovaries of normal and hypogonadal (hpg) mice. J. Mol. Endocrinol..

[22] Kelsey T.W., Wright P., Nelson S.M., Anderson R.A., Wallace W.H. (2011). A validated model of serum anti-mullerian hormone from conception to menopause. PLoS ONE.

[23] Hagen C.P., Aksglaede L., Sorensen K., Mouritsen A., Andersson A.M., Petersen J.H., Main K.M., Juul A. (2012). Individual serum levels of anti-mullerian hormone in healthy girls persist through childhood and adolescence: A longitudinal cohort study. Hum. Reprod..

[24] Lashen H., Dunger D.B., Ness A., Ong K.K. (2013). Peripubertal changes in circulating antimullerian hormone levels in girls. Fertil. Steril..

[25] La Marca A., Malmusi S., Giulini S., Tamaro L.F., Orvieto R., Levratti P., Volpe A. (2004). Anti-mullerian hormone plasma levels in spontaneous menstrual cycle and during treatment with fsh to induce ovulation. Hum. Reprod..

[26] Weintraub A., Margalioth E.J., Chetrit A.B., Gal M., Goldberg D., Alerhand S., Eldar-Geva T. (2014). The dynamics of serum anti-mullerian-hormone levels during controlled ovarian hyperstimulation with gnrh-antagonist short protocol in polycystic ovary syndrome and low responders. Eur. J. Obstet. Gynecol. Reprod. Biol..

[27] Lee J.R., Kim S.H., Kim S.M., Jee B.C., Ku S.Y., Suh C.S., Choi Y.M., Kim J.G., Moon S.Y. (2010). Anti-mullerian hormone dynamics during controlled ovarian hyperstimulation and optimal timing of measurement for outcome prediction. Hum. Reprod..

[28] Andersen C.Y., Byskov A.G. (2006). Estradiol and regulation of anti-mullerian hormone, inhibin-a, and inhibin-b secretion: Analysis of small antral and preovulatory human follicles' fluid. J. Clin. Endocrinol. Metab..

[29] Andersen C.Y., Lossl K. (2008). Increased intrafollicular androgen levels affect human granulosa cell secretion of anti-mullerian hormone and inhibin-b. Fertil. Steril..

[30] Nielsen M.E., Rasmussen I.A., Kristensen S.G., Christensen S.T., Mollgard K., Wreford Andersen E., Byskov A.G., Yding Andersen C. (2011). In human granulosa cells from small antral follicles, androgen receptor mrna and androgen levels in follicular fluid correlate with fsh receptor mrna. Mol. Hum. Reprod..

[31] Liberty G., Ben-Chetrit A., Margalioth E.J., Hyman J.H., Galoyan N., Eldar-Geva T. (2010). Does estrogen directly modulate anti-mullerian hormone secretion in women?. Fertil. Steril..

[32] Gnoth C., Roos J., Broomhead D., Schiffner J., Godehardt E., Freundl G., Johnson S. (2015). Antimullerian hormone levels and numbers and sizes of antral follicles in regularly menstruating women of reproductive age referenced to true ovulation day. Fertil. Steril..

[33] Guerrier D., Boussin L., Mader S., Josso N., Kahn A., Picard J.Y. (1990). Expression of the gene for anti-mullerian hormone. J. Reprod. Fertil..

[34] Grynberg M., Pierre A., Rey R., Leclerc A., Arouche N., Hesters L., Catteau-Jonard S., Frydman R., Picard J.Y., Fanchin R. (2012). Differential regulation of ovarian anti-mullerian hormone (amh) by estradiol through alpha- and beta-estrogen receptors. J. Clin. Endocrinol. Metab..

[35] Couse J.F., Yates M.M., Deroo B.J., Korach K.S. (2005). Estrogen receptor-beta is critical to granulosa cell differentiation and the ovulatory response to gonadotropins. Endocrinology.

